# Construction method for ecological protection of stone side slopes using composite vegetation concrete

**DOI:** 10.1038/s41598-023-43833-0

**Published:** 2023-10-06

**Authors:** Xiying Cheng, Ruimin Yang, Yi Han

**Affiliations:** College of Architecture, Anhui Science and Technology University, Bengbu, 233100 Anhui China

**Keywords:** Forest ecology, Forestry

## Abstract

Traditional ecological restoration technology has many challenges in dealing with the greening of steep rock slopes, especially in the case of serious soil erosion. In order to overcome these problems, this study aims to organically combine the traditional protection and reinforcement technology with the new vegetation restoration technology, and put forward a new ecological protection technology of composite vegetation concrete to realize the comprehensive protection and ecological restoration of rocky slopes. In this paper, by analyzing the mechanism and existing problems of ecological protection of rocky slopes, the design requirements of composite vegetation concrete are studied in detail, and the related construction technology is expounded. In the experiment, the vegetation coverage and incidence were systematically analyzed, and the results showed that some samples showed high vegetation coverage and low incidence. Comprehensive consideration shows that the average vegetation coverage rate is 93.7%, and the average incidence rate is only 5.21%, which all meet the corresponding standards. The composite vegetation concrete technology has a wide application prospect in stone slope protection, which significantly improves the slope vegetation coverage and compressive strength, and effectively promotes the slope greening and ecological sustainable development. Through this study, we aim to convey to readers that the comprehensive method of combining traditional and innovative technologies can achieve encouraging results in solving the problem of ecological restoration of steep rock slopes, and provide useful reference for engineering practice in similar environments.

## Introduction

The composite vegetation concrete ecological protection technology is a new technology that utilizes special concrete formulas and seeds to protect and green mountain slopes. The composite vegetation concrete material has a wide range of raw materials, which not only reduces the construction difficulty, but also ensures the operability principle of side slope protection engineering and the overall effect of greening side slope management. Meanwhile, it solves the shortcomings of traditional side slope protection methods, such as anchor brackets, original paint brackets, support walls, etc., which cannot be used for green side slope support. Composite vegetation concrete materials have excellent corrosion resistance and simple production process^1^. Engineering machinery can truly achieve the perfect combination of side slope support and greening functions by utilizing existing equipment. Therefore, this article explores the construction methods of composite vegetation concrete in side slope protection.

The protection of side slopes is often difficult to construct, with low operability and slow effectiveness. Research on side slope protection helps to find new methods to solve these problems and improve its governance effectiveness. Chi introduced an innovative spiral anchor for horizontal drainage ditches, which was an underground drainage system used for side slope protection^2^. Othman explored the possible success and failure factors of slope protection methods through side slope stability analysis, to determine potential side slope protection schemes for the Sengarang coastal embankment^3^. Nishiwaki further classified the types of side slope protection methods based on rock types or geological time scales from the perspective of representative side slope farmland selected from a hundred selected slope farmland in Japan^4^. Lee discussed the use of alkaline cation crosslinking method of calcium hydroxide and sodium hydroxide to induce non thermal gelation and implemented the site soil with the best performance in the indoor assessment on the site side slope of the abutment^5^. Chun analyzed the main factors of side slope deformation based on the engineering geological conditions and surrounding environment of ancient mining relics and used a deep sliding force monitoring system to continuously monitor the side slope stability of the relics^6^. Liao conducted a case study on airport runway side slopes to provide an optimized design scheme for reinforced soil side slopes with high fill side slopes^7^. Xu systematically explored the impact of local rainy season heavy rainfall on the vast soil side slopes protected by retroviruses in Nanning through in situ comprehensive monitoring and comparative analysis^8^. Fu reviewed the latest research progress in ecological protection technology, identified the main construction processes and types, revealed the protection mechanism of ecological protection technology and analyzed the comprehensive benefits of ecological side slope protection technology^9^. Slope stability protection is to reduce the damage caused by soil erosion, landslide and collapse, and protect land, infrastructure and environment. Vegetation cover, plant root system reinforcement and slope protection net are all common slope stability protection methods. In summary, side slope ecological protection can improve the soil environment of the side slope and increase the vegetation coverage of the side slope soil. However, further research is needed on the application of composite vegetation concrete.

Vegetation concrete has excellent protective effects in side slope engineering and many scholars have analyzed the application of vegetation concrete in side slopes. Ye applied the wet spraying vegetation concrete ecological side slope protection technology to the side slope protection project of Huama Lake water system connecting channel in Ezhou City, Hubei Province and observed the side slope protection and water and soil conservation effects, plant coverage, plant density, average plant height and species diversity indicators on site^10^. Wang explored the similarities and differences in water transport between vegetation concrete and natural soil and found that vegetation concrete could improve the growth effect of plants in side slopes, providing theoretical reference for the irrigation layout and irrigation schedule of soil drip irrigation systems under the ecological restoration of high and steep side slopes^11^. To quantitatively evaluate the erosion resistance of vegetation concrete, which was a typical artificial composite soil, Zhou compared it with natural soil under the same site conditions in the laboratory and determined and calculated the distribution characteristics of aggregate particle size and the protective effect of vegetation concrete on side slopes^12^. In summary, vegetation concrete can significantly improve the growth effect of plants in side slopes, which is of great significance for ecological protection of side slopes.

Composite vegetation concrete is mainly used for greening and protection of stone side slopes. To study the specific application effect of composite vegetation concrete in side slope protection, this article studies the spraying thickness of vegetation concrete in side slope protection and analyzes the spraying thickness under different side slope gradients. In the relevant experimental section, the porosity and pH of the spray layer in side slope protection are studied and it is found that the porosity and pH of the composite vegetation concrete are qualified. Besides, the plant growth rate of side slope protection engineering under composite vegetation concrete has significantly improved and the overall coverage rate of plants has also reached the qualified standard.

## Formula and design of composite vegetation concrete

### Performance design of composite vegetation concrete

The carbon sources required by rhizosphere soil microorganisms in side slopes are mostly composed of sugar water compounds, amino acids and polymers^13^. Perhaps if there is a certain gap at the bottom of the side slope, then under the action of small external forces (such as groundwater, earthquakes, etc.), the side slope experiences instability. In terms of physical properties of composite vegetation concrete, it needs to have good moisture resistance and light resistance and a unit weight of 14–15 kN/m^3^. The porosity is between 30 and 45%. In terms of the mechanical properties of composite vegetation concrete, the strength tested in situ is: 1 month, 0.29 MPa; within three months, 0.38 MPa; 1 year later, 0.42 MPa; two years later, 0.40 MPa; three years later, 0.39 MPa. During the construction of composite vegetation concrete, mesh reinforcement is adopted. Meanwhile, plants planted on the side slope can also effectively resist rainstorm scouring, sun exposure, temperature changes and so on^14^. Besides, its anti-seepage performance is designed based on an average annual precipitation of 2000 ml. In the use of composite vegetation concrete, it is found that the seed germination rate is 90% and the vegetation coverage is 95%. The soil fertility is good and the seed growth status is good.

### Formula design of composite vegetation concrete

The height of high and steep side slopes usually exceeds 30 m and some side slopes do not have raceways. Regardless of the construction measures used, their construction management is very difficult^15^. The same type of composite vegetation concrete, when used in different projects, plays a different role. Therefore, the appropriate mix proportion should be selected based on the actual situation for composite vegetation concrete. The composite concrete side slope protection composed of concrete beams and rock fillers can reduce the overall thickness of the required protective cover^16^. The composite vegetation concrete side slope protection structure is a new type of side slope protection structure. The raw material ratio of composite plant concrete should follow targeted formulas and production principles, so that the formula of composite plant concrete can maximize its function and achieve the maximum effect of side slope protection and greening. The formula and required functions of composite vegetation concrete used in side slope ecological protection are shown in Table 1.

**Table 1:** Formulation and action analysis of composite vegetation concrete.

Constituent materials	Material usage	Role
Loam	82–94%	Ensuring vegetation growth
Cement	5–15%	Improving vegetation intensity and erosion resistance
Organic matter	10–23%	Improving soil structure
Fertilizer	0.5–1.3%	Increasing soil nutrition
Water retaining agent	0.07–0.12%	Enhancing vegetation’s drought resistance ability
Grass seed	35 g	Seeding
Greening admixtures	3–6%	Adjusting soil pH

Usually, sandy loam soil with medium particle size and good three-phase ratio is chosen. The water and fertilizer conditions are good and suitable for plant growth. In composite vegetation concrete, sandy loam is the most important component. At present, measures such as sand molding and adhesive molding have been adopted in side slope protection, but to ensure the healthy growth of plants, it is usually necessary to combine other types of measures for improvement.

Cement has many superior properties and is widely used for side slope protection in civil engineering^17^. Cement, as the main bonding component of composite vegetation concrete, is usually selected as ordinary Portland cement with a requirement for the alkalinity of its clinker not exceeding 0.6%. In the reduction of pH value in composite vegetation concrete, additives such as Portland cement and limestone are used to reduce its pH. After hydration, a mixed vegetation concrete reinforced by a spray layer can be formed, which has a certain strength and can resist rain erosion. The amount of cement added is influenced by the soil pH value, usually ranging from 5 to 12%. If it is too low, it cannot achieve reinforcement effect. If it is too high, it may cause high alkali content, which is unfavorable for plant growth and uneconomical.

In the selection of organic matter for composite vegetation concrete, using fresh rice husks and wood chips, as well as adding decayed rice husks and wood chips as organic matter, can improve its three-phase and water holding properties. Rotten rice husks and sawdust inject a large amount of microorganisms into the composite vegetation concrete. They work together with the plant roots to gradually transform the composite vegetation concrete into soil suitable for plant growth and provide it with certain nutrients such as nitrogen, phosphorus and potassium. Besides, crushed straw can also be used to replace wood chips and rice husks.

In the selection of fertilizers, it is better to combine long term fertilizers with short term fertilizers. Short term fertilizers can promote short-term growth of plants, while long term fertilizers aim to prevent long term degradation of plants.

A series of water retaining agents are used in ecological protection of side slopes: starch series, cellulose series, synthetic polymer series, protein series, other natural product and their derivatives series, mixed series, etc. However, from the perspective of cost-effectiveness, water absorbing resins are commonly used in engineering, which can significantly improve the drought resistance of composite vegetation concrete.

In the selection of grass seed formula for composite vegetation concrete, it is suggested to try to choose imported industrialized grass and shrub seeds and use multiple grass seeds at the same time. In the spraying of composite vegetation concrete, it is necessary to have grass seeds in the upper layer and no grass seeds in the lower layer.

Acid alkali buffering agents, dyes, disinfectants and particulate matter are essential additives in vegetation concrete, which can regulate certain characteristics of composite concrete to stimulate plant growth. For example, adding phosphate plates to flammable industrial waste may have different functions, such as recycling, pH control and improving productivity.

## Construction process for ecological protection of stone side slopes under composite vegetation concrete

The steps of ecological protection of rocky slopes include selecting and matching plant varieties according to slope types and average annual rainfall, thoroughly cleaning rock slopes, gravels and soil of rock slopes, fixing anchor rods, laying iron-zinc nets, preparing composite plant concrete, carrying out high-pressure spraying, and carrying out maintenance and management in the later period.

In the ecological protection of stone side slopes, corresponding protective measures need to be taken according to the specific situation of the stone side slope, as shown in Table 2.

**Table 2:** Ecological protection means for stone side slopes.

Protective measures	Role
Evaluation planning	Develop planning goals for ecological protection
Vegetation cover	Effectively slowing down the rate of soil erosion and increasing the anti erosion ability of the side slope
Vegetation selection	Increasing soil structure and stability
Plant configuration	Forming multi-level root system and vegetation coverage
Drainage system construction	Reducing water erosion and damage to side slopes
Monitoring and maintenance	Maintaining the effectiveness of ecological protection systems
Sustainable management	Continuous management and maintenance

The type of side slope, annual rainfall (duration of drought days) and side slope have a significant impact on the thickness of composite plant concrete spraying. Because the composite vegetation concrete side slope protection and greening technology is mainly applied to stone side slopes and the side slope is usually large and the soil formation speed is lower than the erosion speed, the thickness of the spraying cannot be too small. However, from an economic perspective, it cannot exceed 10 cm. The specific spraying thickness is shown in Table 3.

**Table 3:** Analysis of injection thickness of different stone side slopes.

Side slope type	Average annual rainfall (mm)	Spray thickness (cm)
Hard side slope	500–799	9–10
	800–1299	8–9
	≥ 1300	7–8
Soft side slope	500–799	8–9
	800–1299	7–8
	≥ 1300	6–7

According to Table 3, as the annual precipitation increased, the spraying thickness gradually decreased. This was mainly achieved by increasing the thickness of composite vegetation concrete to compensate for the insufficient water supply in the vegetation soil and improve the soil formation speed of stone side slopes. When the annual average precipitation reached over 1300 mm, the moisture in the vegetation soil recombined and its thickness did not need to be too high. Moreover, under the same precipitation, the spraying thickness of hard side slopes was higher than that of soft side slopes. The reason was that the cracks and weathering degree of stone side slopes could affect the growth of plant roots. Hard side slopes had fewer cracks, so it was necessary to increase the spraying thickness to improve the soil formation rate of hard side slopes and meet the growth of plants.

**Figure 1:**
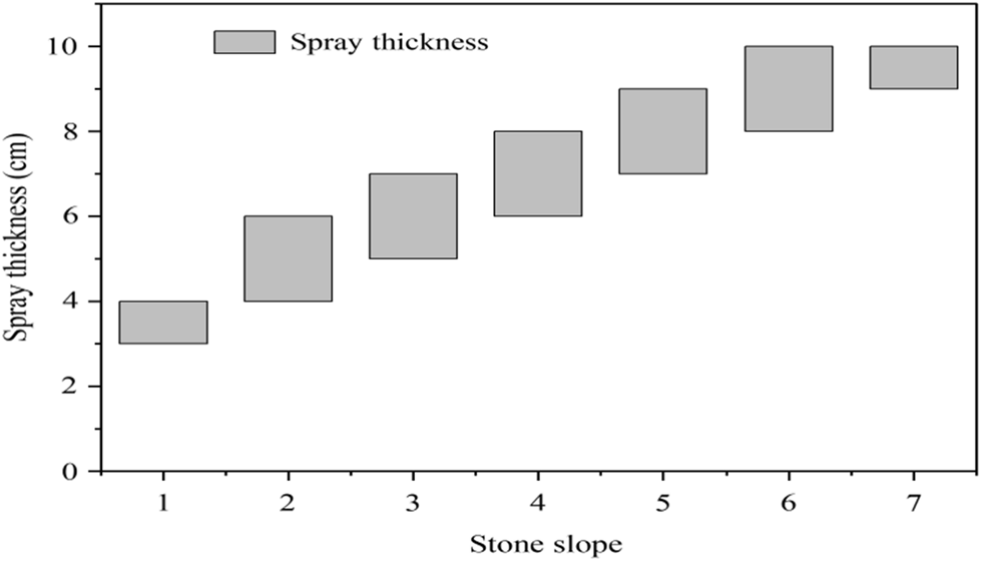
Analysis of spray thickness under different stone side slope gradients.

The factor that affects the spraying thickness is also the side slope gradient, so it is necessary to analyze the spraying thickness of composite vegetation concrete under different gradients. Seven types of side slopes were investigated and their gradients from small to large were stone side slope 1, stone side slope 2, stone side slope 3, stone side slope 4, stone side slope 5, stone side slope 6 and stone side slope 7. The specific spraying thickness is shown in Fig. 1.

In Fig. 1, the x-axis represents different rocky side slopes, while the y-axis represents the spray thickness. According to Fig. 1, as the gradient of the rocky side slope increased, its spraying thickness also gradually increased. Steep slopes had adverse effects on the germination, growth and development of plants, especially in environments with poor soil quality and drought^18^. Under steep slope conditions, water in the soil was easily lost and difficult to store in the soil, seriously affecting the growth of plants in the soil. Therefore, the size of the side slope gradient was closely related to the spraying thickness and the spraying thickness of composite vegetation concrete should be correspondingly increased to improve water conditions and ensure the vegetation growth conditions of the soil. For stable slopes, the compressive strength of the composite concrete plant frame was about 10 MPa.

Plant species have a direct impact on the effectiveness and lifespan of composite vegetation concrete technology. When adopting the ecological side slope protection technology of composite vegetation concrete, the first issue to be paid attention to is the selection and combination of plant species, which must be adapted to the local environment and able to provide sufficient nutrition and water for the vegetation. Trying to choose local plants in the area where the side slope road is located, they are scientifically configured to form the optimal plant community and build a scientific ecosystem. When selecting composite vegetation concrete, the following requirements should be met: adapting to local climate conditions, local types are used as much as possible, achieving diversity of types. The seed germination speed is fast, while the germination rate is high and it has strong drought resistance ability. Combining cold and warm grass seeds can achieve good greening effects. Moreover, after determining the plant variety, a germination test must be conducted with a germination rate of over 95% before subsequent construction can proceed. Based on the specific ecological situation of the side slope, plants with certain cold and drought resistance abilities such as Agropyron cristatum, Medicago sativa, Amorpha fruticosa, Lespedeza bicolor, Caragana korshinskii, Cosmos bipinnatus (all in Latin) and Siberian Elm are selected under low precipitation conditions. Woody plants often choose local tree species and some tree species that are resistant to drought and have strong nitrogen fixation. In cases where the side slope is broken and has many cracks, shrubs can be added to the side slope. For steep slopes with intact rocks, shrubs may not be planted.

After selecting plants, it is necessary to start by completely cleaning the rock side slope, gravel and rock slope soil. It is necessary to clean up dangerous rocks. If the protrusion is strong, tools should be used to adjust the prediction to remove about 50 cm of silt and various joint side slopes, to facilitate the smooth operation of the anchor chain in the future.

In this protection, 12 mm threaded steel bars are selected approximately, arranging a spacing of 1 m × 1 m and selecting a length of 45-65 cm. When anchoring, a hammer can be used to impact it to a depth of 40 cm. During the drilling process, it is necessary to ensure that the hole diameter is 12 mm and that the drilling direction is perpendicular to the slope direction.

In this type of protection, the steel wire mesh used is a diamond mesh of size 4, with a mesh size of 5 cm × 5 cm. In actual paving operations, the distance from the top of the slope is approximately 50 cm. It completely covers the rock side slope from top to bottom. For adjacent mesh holes, parallel docking can also be used to tighten the joints of the mesh holes to form a complete mesh.

According to the actual situation of the side slope, slight adjustments have been made to the cement and additives. Composite vegetation concrete can be divided into two types: the foundation layer and the surface layer. Usually, it is not necessary to add grass seeds to the cement foundation layer. Grass seeds are added on the surface layer, which is convenient to save costs and improve the survival rate of grass seeds. During specific construction, the foundation concrete and surface concrete should be prepared separately and a vertical mixer should be selected for mixing.

Air compressor is selected to conduct high-pressure spray seeding for composite vegetation concrete. The traditional anchor spraying machine should be used from top to bottom, with a thickness of 10–12 cm. Spraying is carried out in two stages, first spraying a base layer of 8–10 cm and then spraying a surface layer of 1–2 cm thick. After high-pressure spraying, composite plant concrete must be cured immediately.

In the proportion of composite vegetation concrete, more consideration should be given to local materials in terms of raw materials to cost reduction and targeted selection of various plants should be combined with the climate characteristics of the project site. The maintenance work of composite vegetation concrete mainly includes three aspects: moisture, fertilizer and prevention of diseases and pests. Firstly, in the germination stage of composite vegetation concrete seeds, it is necessary to control the amount of water sprayed and prevent the water column from directly entering the plant body. Otherwise, it can cause a significant impact on the plant and affect its growth. In high temperatures, plants should not be watered to avoid burning them. Secondly, regular fertilization and sowing should be carried out to ensure that plant seeds receive sufficient nutrition and fertilization should be carried out according to the specific conditions of the plant seeds. Thirdly, the control of pests and diseases is mainly aimed at some common pests, using physical and chemical methods to control them as little as possible to harm the plants and ensure their normal growth.

## Experiment on ecological protection of slope under composite vegetation concrete

### Porosity and pH of spraying layer of composite vegetation concrete

Plants can only grow under specific conditions. In the design of porosity required for plant growth, when the porosity is greater than 20%, it can meet the porosity required for plant growth. Moreover, the larger the porosity, the better the growth of the plant. However, the porosity of composite vegetation concrete can also affect its own mechanical properties. Therefore, multiple factors need to be considered to determine the appropriate porosity. Plants usually live under moderate pH values, but due to the presence of cement, composite vegetation concrete exhibits strong alkalinity in the initial pores, which is not conducive to plant survival. Therefore, changing the alkaline environment in its pores is necessary. Measures such as controlling the amount of cement, adding mineral admixtures and adding a certain amount of acidifiers are usually taken to improve the alkaline environment in the concrete pores.

**Figure 2:**
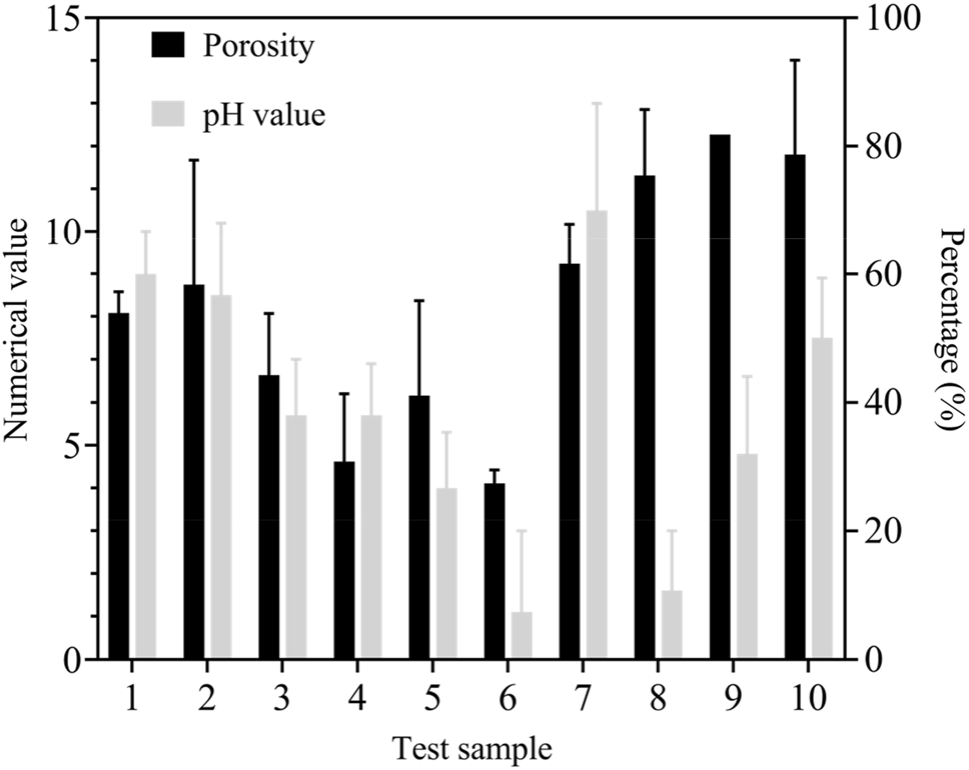
Analysis of side slope porosity and pH under composite vegetation concrete.

The porosity and pH of the spray layer in the ecological protection of side slopes under composite vegetation concrete can affect the growth rate of vegetation. Therefore, this article analyzed the porosity and pH of the spray layer under composite vegetation concrete. In this paper, random sampling was adopted to sample the side slope protection under the composite vegetation concrete. The side slope area was 5000 square meters and one test point was selected for every 500 square meters. In this paper, random sampling method is used to conduct a sampling survey on the site of composite vegetation concrete slope protection. The slope area is 5000 square meters, and a test point is selected every 500 square meters. A total of 10 test samples were selected. The porosity and pH were divided into three grades. The porosity ≤ 25% was unqualified, while 25% < porosity < 45% was qualified and the porosity ≥ 45% was excellent. PH > 9 was considered unqualified, while 8 ≤ pH ≤ 9 was considered qualified and pH < 8 was considered excellent. The specific investigation results are shown in Fig. 2.

The x-axis in Fig. 2 shows the test samples of the side slope, with values on the left y-axis and percentages on the right y-axis. The legend shows porosity and pH from top to bottom, respectively. According to Fig. 2, among the ten side slope samples tested, their porosity was all > 25%. Among them, there were 4 qualified test samples and 6 excellent test samples. In side slope protection, the larger the porosity, the more conducive it was to plant growth in the vegetation, providing a certain growth space for plant root growth, increasing the water absorption of plants and reducing their nutrient needs. From the situation of the test samples, the porosity under plant concrete met the design requirements. Among the tested side slope samples, only one sample had an unqualified pH, while two samples were qualified and seven samples were excellent. The pH often affect the growth of plants, so in the selection of composite vegetation concrete, it is also necessary to reduce its pH to achieve the environment required for plant growth and improve vegetation coverage.

### Effective water holding capacity and compressive strength of side slopes under composite vegetation concrete

The bearing capacity of composite vegetation concrete is mainly transmitted by the bonding point between aggregates. Because the strength of aggregates is high and the bonding area between cement gel and coarse aggregate is small, its failure feature is the destruction of the bonding area of aggregate particles. The composite vegetation concrete side slope protection structure is a new type of side slope protection structure that can reduce the overall thickness of the required protective cover^19^. Therefore, while ensuring a certain porosity, the number and area of connection points can be increased; the strength of the cement layer can be improved; the strength of the composite concrete can be improved. For stable slopes, the compressive strength of the composite concrete plant frame is about 10 MPa. The soil structure of stone side slopes mainly filled with soil has a high ecological protection effect.

**Figure 3:**
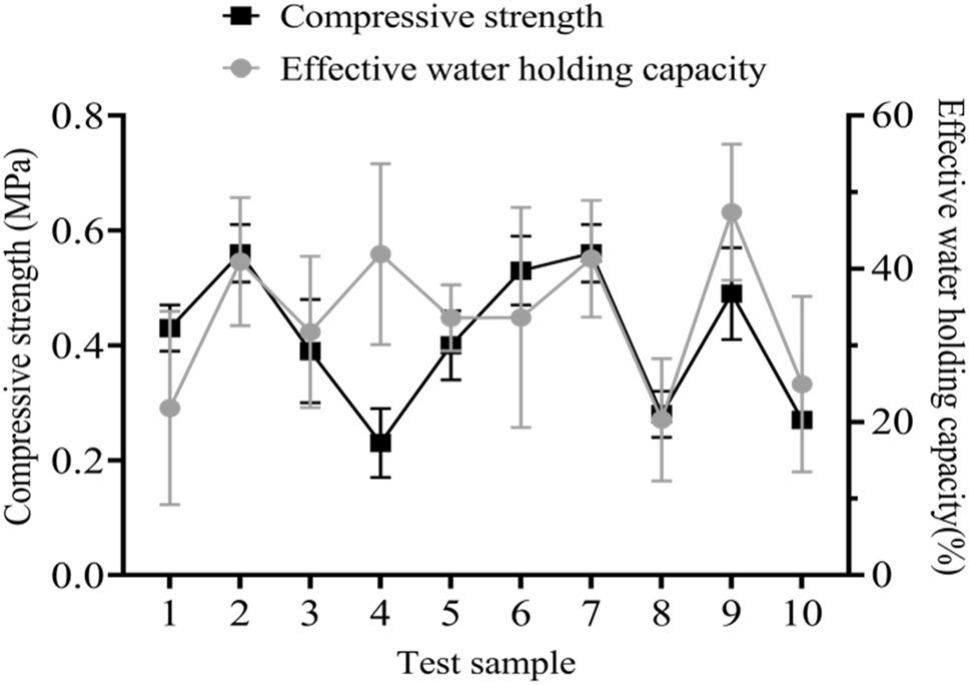
Analysis of effective water holding capacity and compressive strength of side slope.

In side slope protection, the effective water holding capacity of vegetation is a necessary condition to ensure the growth of plants, so the effective water holding capacity and compressive strength of the side slope under the composite vegetation concrete should be analyzed. Similarly, for the side slope of the composite vegetation concrete with an area of 5000 square meters, one test point should be selected every 500 square meters and a total of 10 test samples should be selected, of which the effective water holding capacity and compressive strength were divided into three grades. If the effective water holding capacity was less than 20%, it was unqualified. If the effective water holding capacity was less than or equal to 20%, it was qualified. If the effective water holding capacity was greater than or equal to 30%, it was excellent. Compressive strength < 0.25 MPa was considered unqualified; 0.25 ≤ compressive strength ≤ 0.35 was considered qualified; compressive strength > 0.35 was considered excellent. The specific investigation is shown in Fig. 3.

In Fig. 3, the x-axis represents the test samples of the side slope and the left y-axis represents compressive strength. The right y-axis represents effective water holding capacity. The legends from top to bottom represent compressive strength and effective water capacity, respectively. According to Fig. 3, there were 7 test samples with excellent effective water holding capacity of composite vegetation concrete side slopes, with an average error of 9.73%. Among the excellent compressive strength of side slopes, there were 7 test samples, with a measurement error of 0.05 MPa for compressive strength. In side slope protection, the water holding capacity of composite vegetation concrete needed to provide sufficient water for plant growth within a certain range and the side slope also needed sufficient compressive strength to improve the stability of the batch and prevent vegetation movement and loss on the side slope.

### Vegetation coverage and disease occurrence rate of side slopes under composite vegetation concrete

**Figure 4:**
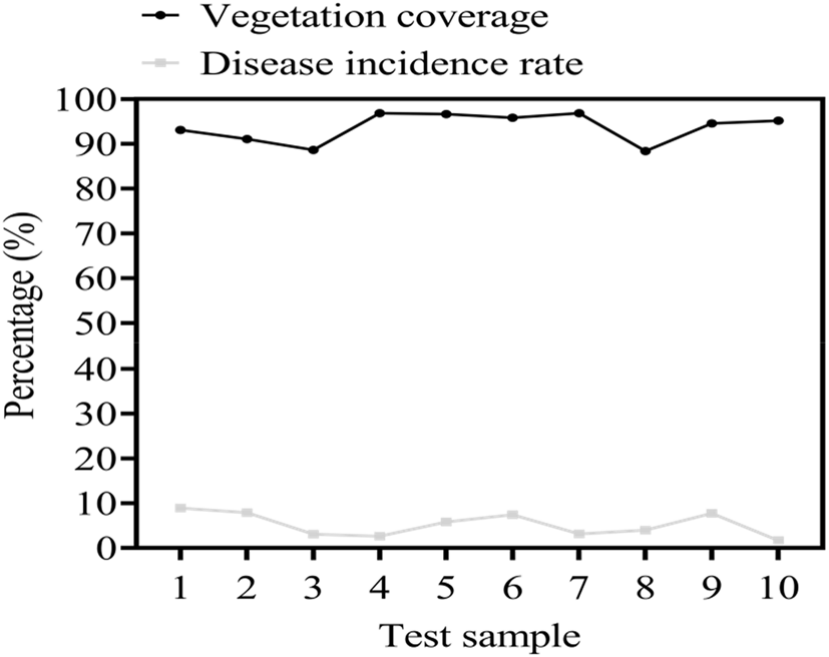
Analysis of vegetation coverage and disease incidence rate of stone side slopes.

In the ecological protection of stone side slopes, vegetation coverage and disease incidence are important criteria for judging their protection effectiveness. This article analyzed the vegetation coverage and disease incidence of a 5000 square meter rocky side slope by extracting one test sample every 500 square meters. The vegetation coverage rate and disease incidence rate were still divided into three levels. Vegetation coverage rate < 85% was considered unqualified; vegetation coverage rate < 95% was considered qualified; vegetation coverage rate > 95% was considered excellent. If the disease incidence rate was greater than 10%, it was considered unqualified; if the disease incidence rate was less than 5%, it was considered qualified; if the disease incidence rate was less than 5%, it was considered excellent. The specific investigation is shown in Fig. 4.

In Fig. 4, the x-axis represents the test samples of the side slope and the y-axis represents the percentage. The legend shows vegetation coverage and disease incidence from top to bottom. According to Fig. 4, there were five test samples with excellent vegetation coverage and five test samples with excellent disease incidence. Overall, the vegetation coverage rate was 93.7% and the disease incidence rate was 5.21%, both of which were qualified. The above data all confirmed the effectiveness of composite vegetation concrete in side slope protection. A high vegetation coverage rate indicated that the plants on the side slope sprouted quickly and had a high germination rate and the greening effect was excellent. The low incidence of diseases indicated that composite vegetation concrete could protect vegetation from the impact of diseases, grow rapidly and ultimately cover the entire side slope to improve its greening effect.

### Evaluation of the overall ecological protection effect of composite vegetation concrete

**Figure 5:**
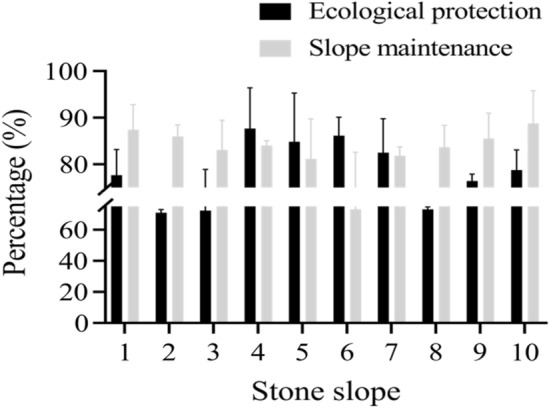
Overall ecological protection and side slope maintenance effect evaluation of side slope under different vegetation concrete.

In the ecological protection of side slopes, vegetation coverage, disease occurrence rate and effective water capacity are the best indicators for evaluating ecological protection^20^. Based on the vegetation coverage rate, disease occurrence rate and effective water capacity investigated in the previous section, the ecological protection and side slope maintenance of side slopes were analyzed. The protective effects of ordinary concrete and composite vegetation concrete were compared and a total of ten side slopes were selected. The specific comparison results are shown in Fig. 5.

In Fig. 5, the x-axis represents the rocky side slope and the y-axis represents the percentage. The legends from top to bottom are ecological protection and side slope maintenance. According to Fig. 5, the ecological protection effect of the side slope under composite vegetation concrete was 5.2% higher than that of ordinary vegetation concrete and the side slope maintenance effect was 5.25% higher than that of ordinary concrete. The stone side slope itself had poor water, fertilizer and soil retention capabilities. To achieve self-restoration of its natural vegetation, the cycle was long and the effect was poor. Under the protection of composite concrete, plants had good contact with soil and rock layers and could draw nutrients from the soil, achieving natural ecological restoration.

### Experimental statement

Experimental research and field studies on plants (either cultivated or wild), including the collection of plant material, it comply with relevant institutional, national, and international guidelines and legislation. Where the grass seeds were obtained?-Purchased.

## Conclusions

Composite vegetation concrete technology organically combines engineering protection with vegetation protection, realizes the sustainable conservation of soil and water, and forms a modular function and an enhanced ecological landscape protection system. It can not only play the role of soil and water conservation, but also restore the ecological environment and strengthen the slope. When selecting composite vegetation concrete, it is necessary to reduce its pH value to achieve the environment needed for plant growth. Slope plants germinate quickly, with high germination rate and good greening effect. Under the protection of composite concrete, plants have good contact with soil and rock layers and can absorb nutrients from soil. In the future ecological protection, it is necessary to further study its raw materials, construction mixture ratio and construction technology. Standardize and standardize, and promote its popularization in ecological protection.

## Data Availability

Data is available upon reasonable request. Please contact the corresponding author if necessary: Xiying Cheng.
